# Evaluation of the geroprotective effects of withaferin A in *Drosophila melanogaster*

**DOI:** 10.18632/aging.202572

**Published:** 2021-01-26

**Authors:** Liubov Koval, Nadezhda Zemskaya, Alexander Aliper, Alex Zhavoronkov, Alexey Moskalev

**Affiliations:** 1 Institute of Biology, Komi Science Centre, the Ural Branch of the Russian Academy of Sciences, Syktyvkar, Komi Republic, Russia; 2 Deep Longevity Ltd, Hong Kong Science and Technology Park, Hong Kong, China

**Keywords:** *Drosophila melanogaster*, lifespan, stress-resistance, stress response, withaferin A

## Abstract

Withanolides are a class of compounds usually found in plant extracts which are an attractive geroprotective drug design starting point. We evaluated the geroprotective properties of Withaferin A (WA) *in vivo* using the *Drosophila* model. Flies were supplemented by nutrient medium with WA (at a concentration of 1, 10, or 100 μM dissolved in ethanol) for the experiment group and 30 μM of ethanol for the control group. WA treatment at 10 and 100 μM concentrations prolong the median life span of *D. melanogaster’s* male by 7.7, 9.6% (respectively) and the maximum life span (the age of death 90% of individuals) by 11.1% both. Also WA treatment at 1, 10 and 100 μM improved the intestinal barrier permeability in older flies and affected an expression of genes involved in antioxidant defense (*PrxV*), recognition of DNA damage (*Gadd45*), heat shock proteins (*Hsp68, Hsp83*), and repair of double-strand breaks (*Ku80*). WA was also shown to have a multidirectional effect on the resistance of flies to the prooxidant paraquat (oxidative stress) and 33° C hyperthermia (heat shock). WA treatment increased the resistance to oxidative stress in males at 4 and 7 week old and decreased it at 6 weeks old. It increased the male’s resistance to hyperthermia at 2, 4 and 7 weeks old and decreased it at 3, 5 and 8 weeks old. WA treatment decreased the resistance to hyperthermia in females at 1, 2 and 3 weeks old and not affected on their resistance to oxidative stress.

## INTRODUCTION

One of the key challenges within life sciences is the search for the substances that can increase the resistance of living systems to various stress factors and contribute to their active longevity. The most promising direction of research in this aspect is the identification of such substances among the plant metabolites. Therefore, the properties of plant extracts are currently actively studied to find the optimal approach to include them in new pharmacological preparations. Among these compounds withanolides are considered as a promising class of candidates for the design of new drugs. Indeed, withanolides display a wide range of relevant pharmacological activities, good bio accessibility and a low risk of side effects. Currently, the preparations containing withanolides from *Withania somnifera* are used in the Ashwagandha composition as a sedative, hypnotic and antiseptic drug [1].

Withanolides are widely studied worldwide. For instance, PubMed contains more than 300 publications with the keyword “withanolides”. Withanolides attract a lot of interest for their potential use as inhibitors of apoptosis. They are also considered as therapeutic candidates for the treatment of neurodegenerative, autoimmune and inflammatory diseases. Their antitumor properties have also attracted a lot of interest for the development of novel cancer therapies. It is common knowledge that *Drosophila melanogaster* has notable advantages as a model system for studying the effects of pharmacological interventions on aging [2]. In our study we hypothesized that the addition of a Withaferin A (WA) supplement to the diet of *Drosophila melanogaster* wild type *Canton-S* (*CS*) could have a beneficial effect on their health status, especially when they get older.

The first withanolide, “withaferin,” was found in the leaves of the *Withania somnifera* (*Solanaceae*) in 1962 [3]. This metabolite was a new type of steroid containing alpha, beta-unsaturated lactone linked to the C-17 of the sterane skeleton [4, 5]. However, this “withaferin” turned out to be 2,3-dihydro-3-methoxywithaferin A, which occurs in mixture with WA [5]. Independently, in 1965 Kupchan et al. found WA in the leaves of *Acnistus arborescens* (*Solanaceae*) [6]. Later, other representatives of this class of compounds were discovered in the plants of the *Solanaceae* family. Withanolides have been found in some *Tacca* species from the *Dioscoreaceae* family (taccanolides) and *Ajuga sp*. from the *Lamiaceae* family, as well as in some marine organisms [7, 8].

**Figure 1:**
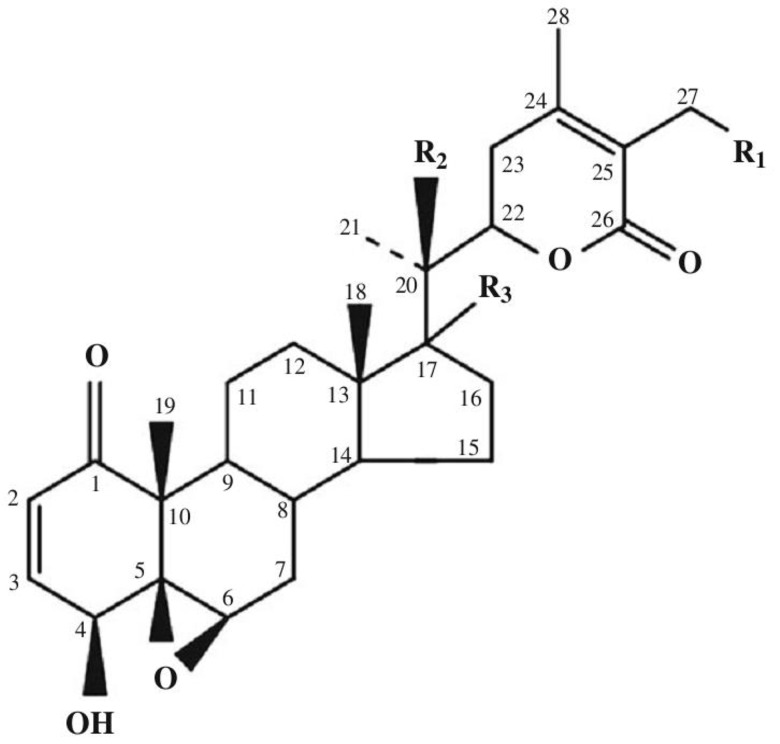
**Basic skeleton of withanolides.** Withaferin A R_1_ = OH, R_2_ = H, R_3_ = H; Withaferin D R_1_ = H, R_2_ = OH, R_3_ = H; 27-Deoxywithaferin A R_1_ = H, R_2_ = H, R_3_ = H; 27-hydroxywithanolide D R_1_ = OH, R_2_ = OH, R_3_ = H; Dihydrodeoxywithaferin A R_1_ = H, R_2_ = H, R_3_ = H; Dihydrowithaferin A R_1_ = H, R_2_ = OH, 17-hydroxywithaferin A, R_1_ = R_3_ = OH, R_2_ = H.

Today, the class of withanolides contains more than 400 chemical compounds. This includes closely related congeners that are found in the plants of *Solanaceae* [9–14]. They consist of C-28 steroidal lactones built on a sometimes modified framework of ergostane, which can form a six-membered lactone ring formed by the oxidation of C-22 - C-26 (Figure 1) [15].

The term “withanolide” is commonly used for 22-hydroxyergostane-26-acid-22,26-olide [4]. Its structural diversity is due to the modifications of the carbocyclic backbone or side chain. The other typical substitutions and modifications of the naturally occurring metabolites are as follows [16]: oxo group at C-1; instead, less commonly a hydroxyl group; double bond C-2 -> C-3; instead, less often the hydroxyl group at C-3; delta-lactone (26 -> 22O), often unsaturated (24, 25); a fragment of gamma-lactone (26 -> 23O) instead of delta-lactone, often also unsaturated; lactol part instead of lactone residue; high oxidation state in many positions of the entire molecule (for example, oxo groups, hydroxyl groups, epoxy substructures, hemicetals); oxidative degradation and or new cyclization of the molecule.

Biosynthesis of withanolides in plants is well studied and proceeds with isoprenoids as precursors [17–19].

WA (4β, 27-dihydroxy-1-oxo-5β, 6β-epoxywitha-2,24-dienolide) (Figure 1) was first isolated from the *Withania somnifera* plant [4]. WA was also found in plants such as *Withania artistata*, *Ajuga bracteosa*, *Vassobia breviflora*, and *Dunalia spinosa* [20–23]. In plants, pure WA is found in relatively small quantities ranging around 0.2-0.3% of dry weight [24].

The stereochemistry of WA was determined in 1966 [25]. Its structure has five functional groups: an unsaturated ketone ring A, 2 hydroxyl groups, an epoxide in ring B, a 6-carbon lactone ring, and an unsaturated carbonyl group (Figure 1). The double bond in ring A and the epoxy ring are responsible for the cytotoxicity of the compound. NMR spectral analysis identified C3 as a major nucleophilic target site for ethyl mercaptan, thiophenol, and ethyl L-cysteine *in vitro* [26]. These five functional groups allow WA to interact with multiple molecular targets leading to a wide range of biological activities.

Previous *in vitro* and *in vivo* studies showed that WA displays anti-tumor activity. It is well established that WA induces apoptosis in cancer cells via different mechanisms [27–30]. In most cancer cell lines, WA inhibits tumor cell proliferation by stopping the cell cycle during the G2/M checkpoint [31, 32] and inhibits nuclear factor kappa B (NF-κB) activation by interacting with the IKKγ subunit, which prevents IκB phosphorylation [33, 34]. A decrease in NF-κB activity leads to a decrease in the production of pro-inflammatory and stress response mediators [35]. Anti-tumor activity is also linked to ability of WA to promote oxidative stress. WA decreases the mitochondrial membrane potential and activates various caspases and proteases, which trigger the degradation of various substrates, such as cytoskeletal proteins and poly (ADP-ribose) polymerase [36, 37]. Also WA regulates the activity of antioxidant enzymes (such as superoxide dismutase) [38] and mRNA expression of antioxidant genes: erythroid 2-like 2 (NFE2L2), heme oxygenase 1 (HMOX1), glutathione-disulfide reductase (GSR), and NAD(P)H quinone dehydrogenase 1 (NQO1)) in cancer cells [39]. Also tumor activity of WA involves induction of heat shock response via Akt / mTOR and MAPK signaling pathways [40].

The anti-inflammatory and anti-fibrotic effects of WA have been demonstrated in an *in vivo* model of bleomycin-induced scleroderma. Daily intraperitoneal injections of WA over the span of 28 days cause reduced dorsal skin thickness in this model. The study has shown that WA suppresses the pro-inflammatory phase of fibrosis regulated by the TGF-β/Smad signaling cascade, and also significantly reduces the proportion of fibroblasts that turn into myofibroblasts. The authors have associated the antifibrotic effect with the inhibition of the FoxO3a-Akt-dependent NF-κβ/IKK-mediated cascade, which is involved in the process of the fibrotic tissue transformation [41].

Due to its wide positive properties and availability, WA can be considered as a promising substance for improving health span and life span. In the present study, our hypothesis is that the addition of WA to the *Drosophila’s* feed would have a beneficial effect on its vitality, especially with age.

## RESULTS

### Effect of WA on the life span of *Drosophila melanogaster* wild type *Canton-S*

**Figure 2:**
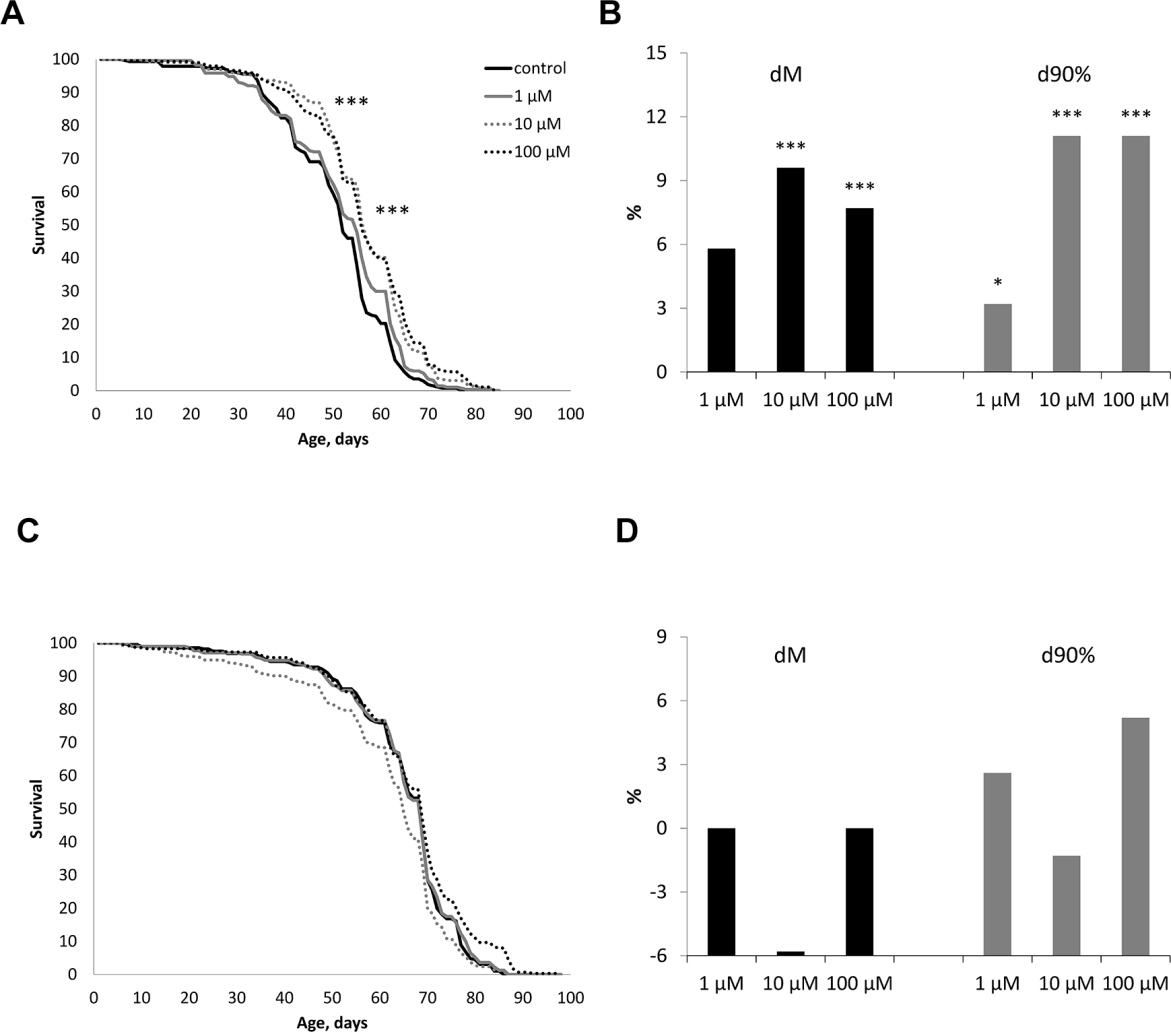
Effect of Withaferin A on the life span in males (**A**, **B**) and females (**C**, **D**) of *Drosophila melanogaster* wild type *Canton-S.* Results of two independent repeats are combined. dM and d90% are the percentage of change in median life span and age of death of 90% of individuals (respectively); * p <0.01, ** p <0.001, *** p <0.0001.

The effect of WA at concentrations of 1, 10, 100 μM on the life span of male and female *Drosophila melanogaster* of the wild type *Canton-S* was studied. WA at 10 and 100 μM concentrations increased the median life span in male by 7.7, 9.6% (respectively) (p<0.0001) and the maximum life span (the age of death 90% of individuals) by 11.1% both (Figure 2B). While we sighted significant shift of these group curves to the right relative to the control curve (Figure 2A). The 1 μM of WA treatment not affected on studied life span parameters. Also WA treatment not affected on studied lifespan parameters in *Drosophila’s* females (Figure 2C, 2D).

### Effect of WA on stress resistance of *Drosophila melanogaster* wild type *Canton-S*

The effect of WA at 1, 10, 100 μM concentrations on the resistance of *Drosophila* to the action of paraquat (20 mM solution in 5% sucrose, oxidative stress) and hyperthermia (33° C, heat shock) at the age of 1 to 8 weeks (from young to old individuals) demonstrated in Tables 1, 2 and Supplementary Figures 1–4.

**Table 1:** Effect of Withaferin A at concentrations of 1, 10, 100 μM on the resistance of Drosophila melanogaster wild type Canton-S males to paraquat (20 mM, oxidative stress) and hyperthermia (33° C, heat shock) at the age of 1 to 8 weeks.

**Age**	**Experimental group**	**Oxidative stress**				**Hyperthermia**			
		**Survival, hours**							
		**25%**	**50%**	**75%**	**90%**	**25%**	**50%**	**75%**	**90%**
1 week	control	64	69	86	92	66	71	81	89
	1 μM	52	67	82	86	63	73	79	83
	10 μM	61	69	79	91	70	81	85	90
	100 μM	48	56	74	86	63	72	81	90
2 weeks	control	41	51	60	73	26	31	33	36
	1 μM	39	52	59	71	28	33	35	41
	10 μM	42	52	62	75	28	32	36	37
	100 μM	42	47	54	71	30	34**	37	40
3 weeks	control	35	40	46	54	18	25	26	27
	1 μM	33	40	47	57	10*	12 ***	14 **	15
	10 μM	32	40	48	56	6 *	7 *	10 *	13
	100 μM	36	42	48	56	21	24	25	27
4 weeks	control	24	30	38	45	20	28	41	45
	1 μM	28 **	34	39	46	29	36	45	50 *
	10 μM	27	32	37	42	27	33	42	47
	100 μM	24	30	37	44	28	33	38	42
5 weeks	control	21	25	31	34	14	18	25	29
	1 μM	19	24	28	35	8 *	11	22	29
	10 μM	21	25	27	31	7	14	20	27 *
	100 μM	23	28	33	37	8 *	12	22	27
6 weeks	control	21	25	30	34	27	32	35	38
	1 μM	19	24	29	33	23	31	39	41
	10 μM	19	23	27	29 *	24	31	40	44
	100 μM	19	25	29	31	27	31	35	38
7 weeks	control	14	20	26	31	10	16	22	26
	1 μM	20 ***	23	29	32	10	14	22	29
	10 μM	16	21	26	30	12	15	23	27
	100 μM	17	20	25	31	9	19	25	33 *
8 weeks	control	13	18	23	27	11	15	20	23
	1 μM	15	20	26	31	10	13 *	17 ***	18 *
	10 μM	14	18	23	28	10	14	17	21
	100 μM	13	17	23	26	9	13	17	20

*Note:* * p <0.01; ** p <0.001, *** p <0.0001. Three biological repeats were combined (32 flies in each). 25%, 50%, 75%, 90% - percentiles.

**Table 2:** Effect of Withaferin A at concentrations of 1, 10, 100 μM on the resistance of Drosophila melanogaster wild type Canton-S females to the action of paraquat (20 mM, oxidative stress) and hyperthermia (33° C, heat shock) at the age of 1 to 8 weeks.

**Age**	**Experimental group**	**Oxidative stress**				**Hyperthermia**			
		**Survival, hours**							
		**25%**	**50%**	**75%**	**90%**	**25%**	**50%**	**75%**	**90%**
1 week	control	64	69	86	92	64	73	77	81
	1 μM	52	67	82	86	60	73	81	88
	10 μM	61	69	79	91	52*	61*	71	76
	100 μM	48	56	74	86	63	72	88	89
2 weeks	control	48	72	103	116	28	38	42	47
	1 μM	47	70	95	111	25	34	42	51
	10 μM	55	77	95	111	22	34	37	42
	100 μM	48	78	97	108	19	28**	34	42
3 weeks	control	42	64	79	99	25	30	35	38
	1 μM	32	50	75	93	15 ***	17 ***	20 ***	23
	10 μM	48	66	82	104	11 ***	13 ***	16 ***	18
	100 μM	40	62	76	95	24	29	33	38
4 weeks	control	28	49	60	78	24	31	36	41
	1 μM	30	48	58	82	26	33	37	43
	10 μM	31	45	67	85	26	31	38	40
	100 μM	30	47	68	82	28	34	41	44
5 weeks	control	28	31	43	50	15	21	24	29
	1 μM	27	42	48	61	15	19	23	26
	10 μM	29	36	45	63	18	22	25	28
	100 μM	27	31	41	48	14	18	22	24
6 weeks	control	21	27	34	57	20	25	31	35
	1 μM	20	26	39	55	22	26	30	33
	10 μM	19	26	36	61	21	23	27	31
	100 μM	21	26	37	51	22	27	30	36
7 weeks	control	19	25	34	44	15	21	26	29
	1 μM	22	25	36	48	13	18	24	27
	10 μM	17	23	30	45	15	19	24	29
	100 μM	19	23	33	42	14	18	23	29
8 weeks	control	17	23	28	37	8	14	19	26
	1 μM	14	22	30	48	9	14	21	25
	10 μM	17	22	30	41	9	14	20	24
	100 μM	18	23	31	40	10	15	20	24

*Note:* * p <0.01; ** p <0.001, *** p <0.0001. Three biological repeats were combined (32 flies in each). 25%, 50%, 75%, 90% - percentiles.

WA treatment had a different effect in response to studied stressors in males. It increased the resistance to paraquat only at the age of 4 and 7 week at 1 μM (by 16.7, 42.9 %% increased 25 percentiles respectively). At 6 weeks decreased 90 percentiles by 14.7% at 10 μM of WA. Also we observed shift in mortality distribution curves at 1 μM of WA in 1 and 8 weeks relative to the control curve. WA treatment increased the resistance of male’s to hyperthermia at 2 weeks after 100 μM of WA (by 9.7% increased 50 percentiles), at 4 weeks after 1 μM of WA (by 11.1% – 90 percentiles) and at 7 weeks after 100 μM of WA (by 26.9% – 90 percentiles). While it decreased the resistance to hyperthermia at 3 weeks after 1 and 10 μM (by 44.4, 52.0, 46.2%%; 66.7, 72.0, 61.5 %% decreased 25, 50, 75 percentiles respectively). At the age 5 weeks we found decreased by 42.9% in 25 percentiles after 1, 100 μM and by 6.9% in 90 percentiles after 10 μM WA treatments. And at the age 8 weeks decreased by 13.3, 15.0, 21.7 %% in 50, 75, 90 percentiles after 1 μM of WA. These data confirm by mortality distribution. We observed shift in mortality curves at 1 μM of WA in 3, 4 and 8 weeks, at 10 μM in 3 and 5 weeks, and at 100 μM at 7 and 8 weeks relative to the control curve. In other variants of the experiment WA not affected on studied survival parameters (Table 2 and Supplementary Figures 1, 2).

WA treatment not affected on female’s resistance to paraquat and reduced resistance to hyperthermia. At the age of 1 week at 10 μM (by 18.8, 16.4 %% decreased 25 and 50 percentiles respectively), of 2 weeks at 100 μM (by 26.3 %– 50 percentiles) and of 3 weeks for 1 and 10 μM (40.0, 43.3,42.9; 56.0, 56.7, 54.3%% - 25, 50 and 75 percentiles respectively). Also these data confirm mortality distribution. We observed shift 100 μM at 2 weeks and 1 μM and 10 μM at 3 weeks curves to the right relative to the control curve. In other variants of the experiment WA not affected on studied survival parameters (Table 2 and Supplementary Figures 3, 4).

### Effect of WA on the intestinal barrier permeability of *Drosophila melanogaster* wild type *Canton-S*

Changes in the permeability of the intestinal barrier in *Drosophila melanogaster* wild type *Canton-S* at the age of 4, 6, 8 weeks were studied against the background of treatment of WA at concentrations of 1, 10, 100 μM.

**Figure 3:**
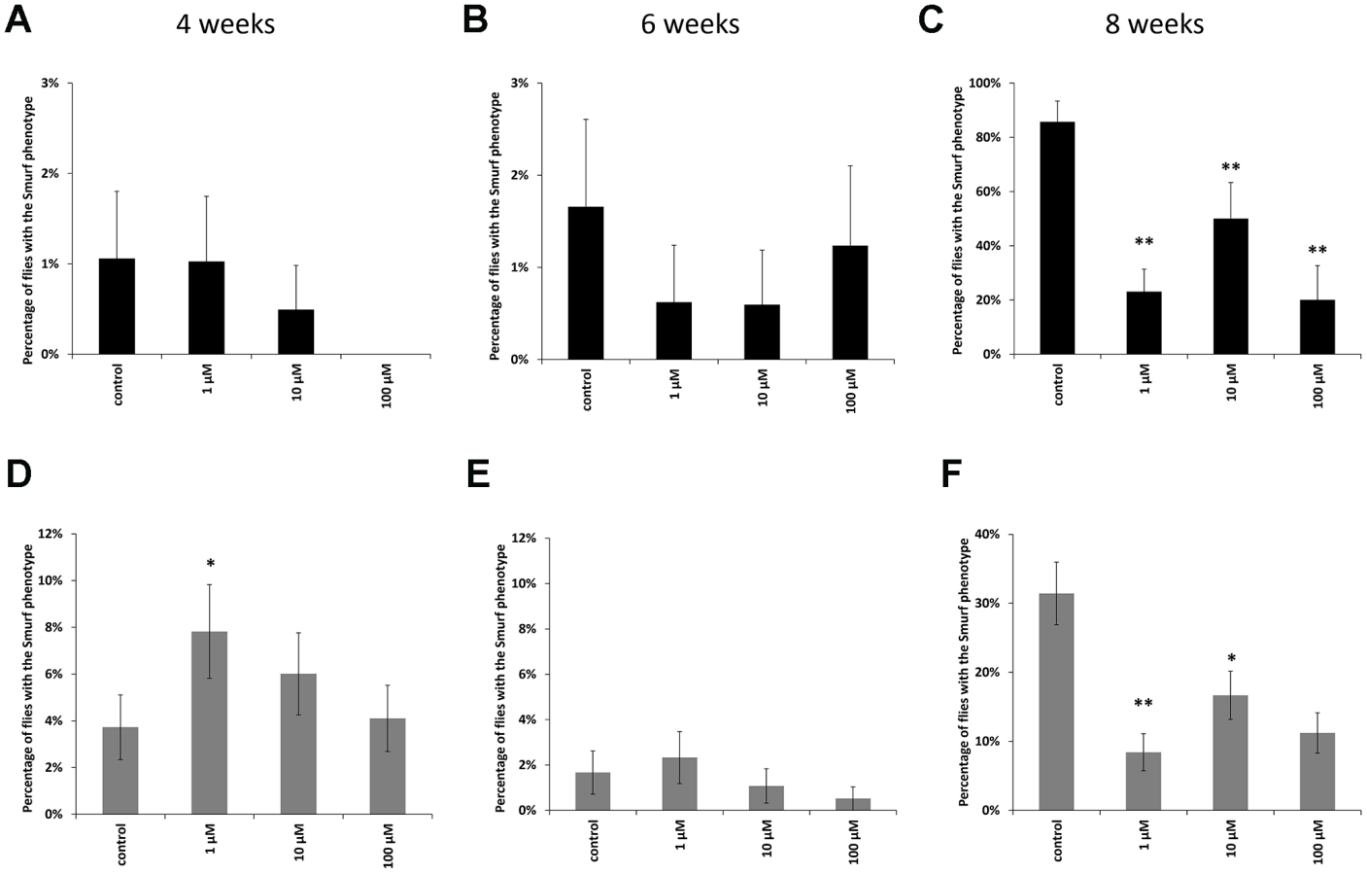
Results of the intestinal barrier permeability test (smurf test) in males (**A**–**C**) and females (**D**–**F**) of *Drosophila melanogaster* wild type *Canton-S* at the age of 4, 6 and 8 weeks. * p <0.01, ** p <0.001.

In males at the age of 8 weeks, a decrease in the proportion of flies with the «smurfs» phenotype by 67, 61 and 89% was observed relative to the control when taking WA at concentrations of 1, 10, 100 μM, respectively (Figure 3C). Females also showed a decrease in the «smurfs» rate only at the age of 8 weeks. The proportion of flies with the «smurfs» phenotype was lower by 73, 42 and 61 %% relative to the control after WA at 1, 10, 100 μM concentrations, respectively (Figure 3F). Other group of the experiment showed no significant differences (Figure 3A, 3B, 3D, 3E).

### Effect of WA on the expression of stress response genes in *Drosophila melanogaster* wild type *Canton-S*

**Figure 4:**
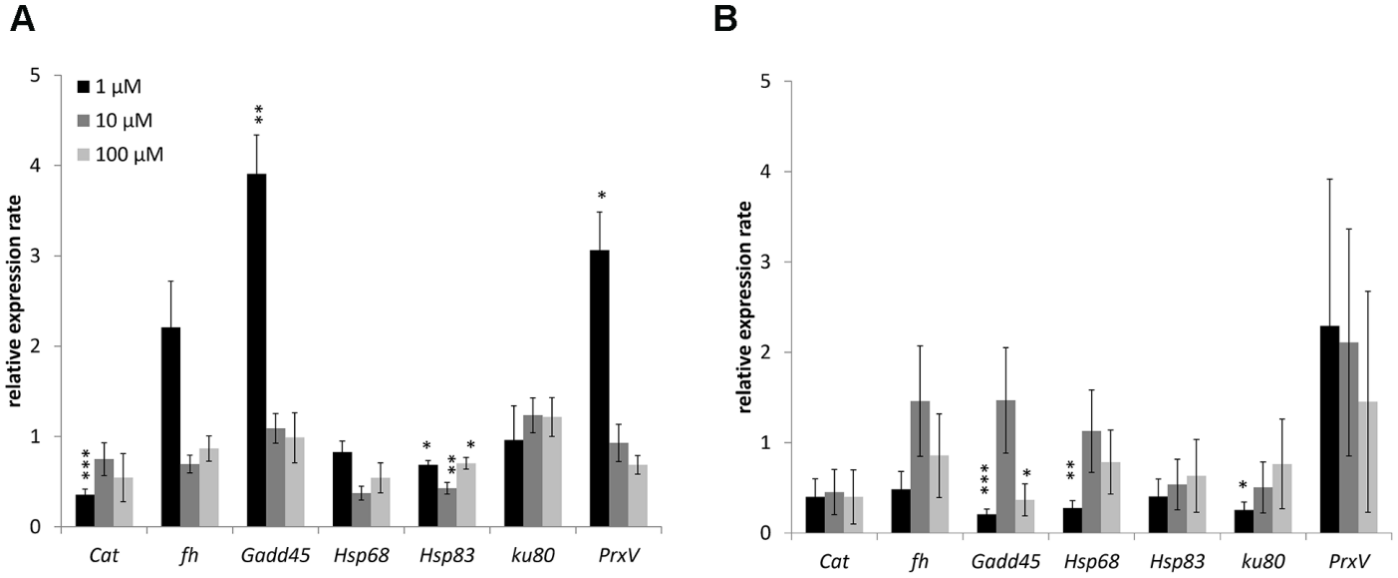
Changing of stress response genes expression in males (**A**) and females (**B**) of *Drosophila melanogaster* wild type *Canton-S* after WA treatment. Results were normalized to control. * p <0.01, ** p <0.001, *** p <0.0001.

Changes in the expression of genes involved in antioxidant defense (catalase, Peroxiredoxin V), metal detoxification (frataxin), recognition of DNA damage (Gadd45), heat shock proteins (Hsp68, Hsp83), and repair of double-strand breaks (*Ku80*) studied. The combined results are presented in Figure 4 and considered. In males 1 μM WA effected on expression of *Gadd45* (3.9-fold-increase)*, Hsp83* (1.5-fold-decrease) and *PrxV* (3.1-fold-increase) genes. The10 μM and 100 μM of WA decreased the expression of only *Hsp68* (2.3-, 1.4-fold respectively) gene (Figure 4). In females the 1 μM of WA treatment decreased the expression of *Gadd45* (4.9-fold)*, Hsp68* (3.6-fold)*, Ku80* (3.9-fold) genes. The100 μM WA treatment decreased the expression of only *Gadd45* (2.7-fold) gene (Figure 4). The expression of other studied genes was not significantly changed.

## DISCUSSION

Currently, the identification of geroprotectors which could be used as a cure against aging constitutes an important area of research. Although there are around 400 compounds known to extend the life span of model organisms, only few of them meet the criteria to be used as potential geroprotectors [42]. Moreover, there is a lack of clinical studies which have been conducted to analyze the effects of these potential geroprotectors on humans. It should be emphasized that an ideal geroprotector should not only increase the average but also the maximum life span. Furthermore geroprotectors should contribute to shifting the entire mortality curve to the right so that extended life span would be associated with the extension of the active period of life.

Balanced nutrition is one of the most important factors promoting increased life span. Currently, rapamycin, metformin, Skulachev ions (SkQ), and some other compounds are known to be promising geroprotective substances [43–46]. The data available indicate that these geroprotective substances prolong the life in model organisms (*Caenorhabditis elegans*, *D. melanogaster*, *Mus musculus*, *Rattus norvegicus*, etc.). In many cases they also reduce the likelihood of aging-associated diseases. Metformin and rapamycin are two FDA-approved mTOR inhibitors. However, the use of metformin and rapamycin has various side effects. A. Aliper et al. [47] applied several bioinformatic approaches and deep learning techniques to a dataset from the Library of Integrated Network – based Cellular Signatures (LINCS) to find the substances that could emulate the genetic response to metformin and rapamycin. Using this approach, the authors predicted the safety of each selected compound. As a result of the analysis, many new candidate mimetics of metformin and rapamycin were identified, WA being one of them.

### Effect of WA on the life span of *Drosophila melanogaster* wild type *Canton-S*

Life span is regulated by multiple interrelated phenotypic and genotypic factors and is a temporal characteristic of the damage-restoration process in the body, leading to old age and death [48–50]. In gerontology, it is important to make a distinction between chronological and biological age. Chronological age is measured as the period passed since the time of birth. In humans, chronological age is not a sufficient metric to evaluate the health and performance of an aging person. A more appropriate measure to this end is the biological age which aims to estimate how the progressive degradation occurring within the aging organism affect a combination of metabolic, structural, functional, regulatory features, and adaptive capabilities. These alterations affect vital functions of the organism, leading to the onset of age related diseases, an increase in the probability of death, or a decrease in life span [51–53]. It is well known that it is possible to delay aging and the onset of age-related diseases to prolong the period of active life. In our study, we have shown that WA at concentrations of 10, 100 μM increases the median and maximum life span of male *CSs* (Figure 2A, 2B). While WA treatment not affected on life span parameters in *Drosophila’s* females (Figure 2C, 2D).

### Effect of WA on stress resistance of *Drosophila melanogaster* wild type *Canton-S*

There are a lot of articles in which plant materials have a positive influence on stress resistance of model organisms. For example it was shown in *Drosophila melanogaster* that the apple phlorizin [54], cloudberry extract [55], oil from *Caryocar coriaceum* (Pequi) [56] increase resistance to oxidative stress. Blueberry extract [57], *Lonicera japonica* extract [58] promote stress tolerance in *Caenorhabditis elegans. Styphnolobium japonicum* fruits [59], *Rhodiola rosea* root extract [60], *Cotinus coggygria* extract [61] increase stress resistance and exert antioxidant properties in mouse models. Therefore, we decided to check the effect of WA on the resistance of Drosophila’s flies to oxidative stress and hyperthermia.

Oxidative stress is caused by elevated intracellular levels of reactive oxygen species (ROS), which damage lipids in cell membranes, oxidize proteins, and damage DNA [62]. In our experiment we use paraquat as inductor of ROS [63]. It is known that WA can suppress oxidative stress [64–67]. In most variants of our experiment, WA not led to significant changes on studied survival parameters. WA treatment at 1 μM increased the resistance of males only at the age of 4 and 7 week. Negative effect was found after 10 μM treatment at the age 6 weeks. Also we observed shift in male’s survival curves at 1 μM of WA in 1 and 8 weeks relative to the control curve (Table 1 and Supplementary Figure 1). While WA treatment not affected on female’s resistance to paraquat (Table 2 and Supplementary Figure 3).

Prolonged or intense heat shock causes numerous changes in cell metabolism and disrupts the state of its structural units [68, 69]. Protein damage is the main type of damage during heat shock. Its downstream effects, higher metabolic rate and free radical production, lead to consequent DNA damage [70–72]. In our experiment, WA treatment had different effect in response to hyperthermia. Thus, it increased the male’s resistance to hyperthermia at 2 and 7 weeks after 100 μM of WA and at 4 weeks after 1 μM of WA. It decreased its resistance to hyperthermia at 3 weeks after 1 and 10 μM, at 5 weeks after 1, 10 μM, 100 μM and at 8 weeks after 1 μM of WA. Also we observed shift in male’s mortality curves at 1 μM of WA in 3, 4 and 8 weeks, at 10 μM in 3 and 5 weeks, and at 100 μM at 7 and 8 weeks relative to the control (Table 1 and Supplementary Figure 2). WA treatment reduced female’s resistance to hyperthermia at the age of 1 week after 10 μM, of 2 weeks after 100 μM and of 3 weeks after 1 and 10 μM. Also we observed shift 100 μM at 2 weeks and 1 μM and 10 μM at 3 weeks curves to the left relative to the control curve (Table 2 and Supplementary Figure 4). Thus, it has been shown that WA has a multidirectional effect on the resistance of *CSs* to the stress factors under study.

### Effect of WA on the intestinal barrier permeability of *Drosophila melanogaster* wild type *Canton-S*

The gastrointestinal tract has a barrier function that prevents the penetration of food antigens, bacterial toxins, viruses and microorganisms into circulation [73, 74]. There are a lot of articles that have been written about positive role of plant extracts on intestinal microflora and intestinal epithelial barrier [75–79]. The disadvantage of these studies is that they were performed *in vitro* and do not take into account the effect of aging. The deregulation of the barrier function, which typically occurs in the elderly, can cause the development of pathological conditions [65, 80–82]. In order to prevent the development of such conditions, methods for diagnosing the violations of the permeability of the intestinal barrier are being intensively developed. Therefore, an analysis of the permeability of the intestinal barrier was performed. We have shown that WA at all studied concentration increases the strength of the intestinal barrier in old *CSs* (Figure 3C, 3F). The rate of flies with the «smurfs» phenotype was lower by 67, 61 and 89% relative to the control after WA at 1, 10, 100 μM concentrations, respectively in males, and by 73, 42 and 61 %% respectively in females. WA’s effects on the strength of the intestinal barrier have not been found in literature. But there is study in which authors did not succeed to increase the strength of intestinal barter using plant materials in aging aspect: ursolic acid (triterpenoid) does not affect gut integrity in male *D. melanogaster* at the age 30 days [83]. And pectin supplementation was not affected by the intestinal barrier function in healthy young adults and in healthy elderly [83].

### Effect of WA on the expression of stress response genes in *Drosophila melanogaster* wild type *Canton-S*

Genetic and epigenetic mechanisms and genes that are involved in the regulation of life span are highly interconnected and related to stress response [50]. Moreover, the overexpression of longevity genes listed in [84] as stress response genes almost exclusively resulted in life span extension. A wide-scale comparative analysis of the 1805 known longevity-associated genes across 205 species disclosed that these genes are consistently overrepresented across diverse taxa, compared with the orthologs of other genes, and this conservation is highly. Also in that study it was shown that longevity-associated genes were enriched in translational processes, energy metabolism and DNA repair genes [84]. The genes analyzed in our study play important roles in the following molecular and biological processes: antioxidant defense (*Cat, PrxV)*, metal detoxification (*f*h), heat shock response (*Hsp68, Hsp83*), DNA damage recognition *(Gadd45)* and double-strand break repair (*Ku80*). More detailed information of these genes can be found in Supplementary Table 1.

Here in *Drosophila’s* male WA decreased heat shock proteins *(Hsp68 or Hsp83)* genes expression at all concentration and increased *Gadd45* and *PrxV* genes expression at 1 μM of treatment. In *Drosophila’s* female found decreased expression of *Gadd45, Hsp68* and *Hsp83* genes after 1 μM of WA. The 100 μM of WA treatment decreased expression of only *Gadd45* gene.

The effects of plant materials on gene regulation have been shown in numerous experiments on model organisms and cancer cell lines. It was shown that licorice and orange extract provoke enhancement of catalase activity and also extend *Caenorhabditis elegans* life span [85, 86]. Citrus and apple pectin’s have induced the expression of genes involved in DNA repair (*D-Gadd, mei-9, spn-B*), apoptosis (wrinkled/hid) and heat shock response (*hsp70Aa*) in *Drosophila* [87]. Overexpression of *PrxV* gene can abrogate shikonin-induced cell apoptosis in HT29 colon cancer cells [88]. Modulation of *HSP 90* and *HSP 70* genes expressions is a possible mechanism by which the *Flueggea leucopyrus* (Willd) decoction mediates cytotoxic effects in breast cell lines [89]. Anticancer property has also been studied in *Glycyrrhiza glabra* which inhibited proliferation in HT-29 cell line due to down-regulation of *HSP90* gene expression which implied an ability to induce apoptosis [90]. Crude phenolic extracts from extra virgin olive oil directly up-regulated the expression of the *Gadd45* gene family in JIMT-1 human breast cancer cell line that circumvent breast cancer resistance to *HER1/HER2*-targeting drug [91]. Protective role of *Podophyllum hexandrum* rhizomes and *Myrtus communis* leaves against DNA damage proved. Shown significant up-regulation of *DNA-PKcs* and *Ku80* and downregulation of *ATM* and *53BP1* gene expressions in cell lines which were pre-treated with mixture of three active derivatives isolated from the rhizomes of *Podophyllum hexandrum*, and then irradiated [92]. Myricetin-3-o-galactoside and myricetin-3-o-rhamnoside, isolated from the leaves of *Myrtus communis*, modulated the expression patterns of cellular genes involved in DNA damaging repair (*XPC, LIG4, RPA3, PCNA, DDIT3, POLD1, XRCC5, MPG*) [93].

We have repeatedly observed gender specific reactions to WA treatment. Individuals of different genders can response differently to dietary restriction and distorted activity of nutrient-sensing pathways [94]. The main pathways and interventions that lead to sex-specific life span responses, include the growth-hormone/insulin-like growth factor 1 (GH-IGF1) axis, mechanistic target of rapamycin (mTOR) signaling, and nutritional and pharmacological interventions [95].

Thus, WA at concentrations of 10, 100 μM increases the median and maximum life span and shifts the curve to the right side in *Drosophila’s* male. Together with WA at all concentration decreased expression of genes involved in heat shock response (*Hsp68* or *Hsp83*). The 1 μM of WA increased expression of DNA damage recognition (*Gadd45*) and antioxidant (*PrxV*) genes. WA treatment had no effect on life span parameters in *Drosophila’s* females. While 1 μM and 100 μM of WA decreased the expression of *Gadd45* gene. And 1 μM of WA also decreased the expression of *Hsp68* and *Ku80* (double-strand breaks repair) genes. WA has also a multidirectional effect on the stress resistance of flies. The 1 μM of WA treatment increased the male’s resistance to oxidative stress only at 4 and 7 week old. Negative effects were found after 10 μM treatment in males at the age 6 weeks, while WA treatment did not affect the female’s resistance to oxidative stress. WA increased the male’s resistance to hyperthermia at 2 and 7 weeks after 100 μM of WA and at 4 weeks after 1 μM of WA. The 1 μM of WA decreased male’s resistance at 3, 5 and 8 weeks old. The 10 μM of WA decreased it resistance at 3 and 5 weeks old. Also 100 μM of WA decreased male’s resistance at the age 5 weeks. WA treatment reduced female’s resistance to hyperthermia at the age of 1 week after 10 μM, of 2 weeks after 100 μM and of 3 weeks after 1 and 10 μM. In contrast to this WA increases the permeability of the intestinal barrier of old flies both sexes.

## MATERIALS AND METHODS

In our study, we used the wild type strain *Canton-S* (Bloomington, USA) *Drosophila melanogaster (CS)*. All *CSs* were kept in Binder climate chambers (KBF720-ICH, 720l, Binder, Germany) at 25° C and a 12-hour illumination regime in 40ml tubes with 5ml of nutrient medium [96, 97].

To obtain the experimental *CS* flies, their parents were pre-planted in jars of nutrient medium in the amount of 10 pairs per tube and left for 24 hours to lay eggs. After the appearance of imago, flies were anesthetized using CO2 anesthesia (Genese Scientific, USA), were separated by sex and were placed in test tubes with nutrient with WA and without for further experiments. Non-virgin females were used. Males and females lived separately with 30 animals per tube. From day 1 of life, 30 μl of of 1, 10 or 100 μM WA ethanol solution on top of the flies’ nutrient medium instilled. As a control, we used flies fed with a medium supplemented with 30 μl of ethanol. The final concentration of the drug in the media was 1, 10 or 100 μM. This concentration has shown its ability to increase life span in human fibroblast (internal preliminary tests) and represents a suitable concentration range for pro-longevity effects in invertebrates. To maintain these concentrations flies were transferred to a fresh nutrient medium twice a week [98–102].

### Life span assay

To assess life span, 150 flies were selected for each experiment in a single repetition. Two biological repeats were made. Combined data are presented. Flies were placed in test tubes with nutrient medium (30 animals per tube). The counting of the number of dead flies was performed daily. The data were used to compute survival curves and the median, maximum life span, 90% death time were calculated. The Kolmogorov-Smirnov test was used to compare the distribution of mortality in survival curves and the Gehan-Breslow-Wilcoxon test was used to compare the differences in median life span. The significance of the differences in maximum life span was evaluated using the Wang-Allison test. In order to apply this method, animals in each variant of the experiment were divided into two groups: with a life span above the age of 90% mortality, or below the age of 90% mortality. Data were recorded in a 2x2 contingency table and compared using the chi-squared test. According to Bonferroni correction were considered significant differences at p less than 0.017. Analyses were performed using Statistica 6.1 (Stat Soft), and online application for survival analysis «Oasis2» (Structural Bioinformatics Lab).

### Stress resistance analysis

The stress resistance of the flies was assessed every week up to 8 weeks of age. The DAM (Drosophila Activity Monitor) system (TriKinetics, USA) was used to look into stress resistance. For analyzing the resistance to oxidative stress, the flies were placed in glass tubes 5 mm in diameter with 20 mM paraquat (Methyl Viologen, Sigma) in 5% sucrose and kept at 25° C until the complete death of flies’ cohort. To assess the resistance to hyperthermia, the flies were seated in glass tubes 5 mm in diameter with a standard medium and kept at 33° C until the complete death of flies’ cohort. The data were used to compute differences in survival distribution with age and in percentiles (25, 50, 75, 90) of death. Fisher’s exact test was used to calculate the statistical differences in percentiles of death. Log-rank criteria were used to assess the statistical significance in survival function distribution. Data were computed using «Oasis2» (Structural Bioinformatics Lab). According to Bonferroni correction were considered significant differences at p less than 0.017. The experiment was performed in three biological repetitions (32 flies in each).

### Smurf test

We used 100 flies per variant of experiment. The smurf test was performed at 4, 6 and 8 weeks of age. For this, the test tubes were prepared with a nutrient medium stained with 2.5% (w / v) blue dye No. 1 (Sigma Aldrich, USA). The flies were moved to this medium for 9 hours. Then the number of «smurfs» and «non-smurfs» was counted. Flies were considered «smurfs» if they were blue outside the digestive system [103]. The data obtained were used to construct histograms of the distribution of «smurf» proportion in samples. Fisher’s exact test was used to assess the statistical significance of differences at p less than 0.017 using «Oasis2» (Structural Bioinformatics Lab).

### Analysis of stress response gene expression

For each variant of the experiment, 60 flies were selected, separated into two groups of 30 and kept under standard conditions, (Genesee Scientific, USA). For each point of the experiment, 10 females and 20 males were used. Expression analysis was carried out 10 days after the emergence of adults. The experiment was carried out in two biological and three analytical replicates.

Gene expression was measured by “real-time” quantitative PCR with a reverse transcription step (RT-qPCR). RNA was isolated using an Aurum Total RNA mini kit (Bio-Rad, USA) according to the manufacturer’s instructions. The concentration of the resulting RNA was measured using a Quant-iT RNA Assay Kit (Invitrogen, USA). Next, cDNA was synthesized according to the iScript cDNA Synthesis Kit (Bio-Rad, USA). The reaction mixture for carrying out the PCR reaction was prepared according to the manufacturer’s instructions iTaq Universal SYBR Green Supermix (Bio-Rad, USA) and primers (Lumiprobe, USA) (Table 3). The polymerase chain reaction was carried out in a CFX96 amplifier (Bio-Rad), with a DNA melting step using the following program: 1) 95° C for 30 s, 2) 95° C for 10 s, 3) 60° C for 30 s, 4) steps 2-3 were repeated 40 times, 5) DNA melting cycles.

**Table 3:** List of studied genes and their nucleotide sequence primers.

**Target gene**	**Abbreviation**	**5’- 3 ‘sequences of forward / reverse primers**
eukaryotic translation elongation factor 1 alpha 2	*eEF1α2*	AGGGCAAGAAGTAGCTGGTTTGC/ GCTGCTACTACTGCGTGTTGTTG
β-Tubulin at 56D	*Tubulin*	GCAACTCCACTGCCATCC/ CCTGCTCCTCCTCGAACT
Ribosomal protein L32	*RpL32*	GAAGCGCACCAAGCACTTCATC/ CGCCATTTGTGCGACAGCTTAG
Catalase	*Cat*	CCCAAGAACTACTTTGCTGAGGTG/ AGGAGAACAGACGACCATGCAG
frataxin	*fh*	TTACAGCGATGGCGTGCTAACC/ AGTGCCGACGAAATCGTATCGC
Growth arrest and DNA damage-inducible 45	*Gadd45*	GCAAACGCACAACCAAAC/ GGCCATCAGGCAGAAGAG
Heat shock protein 68	*Hsp68*	TGGGCACATTCGATCTCACTGG/ TAACGTCGATCTTGGGCACTCC
Heat shock protein 83	*Hsp83*	AAGATGCCAGAAGAAGCAGAGACC/ ATCTTGTCCAGGGCATCGGAAG
Ku80	*Ku80*	GAGCTTCAGAATGTCGCAACTACC/ GGAAAGTCGTTGAAATCGAAGAGC
Peroxiredoxin V	*PrxV*	CCGATGAGCTGAAGTCCAAG/ TTGCCGTTCTCCACCACCAG

The expression of studied genes calculated relatively to the expression of the housekeeping genes *Tubulin, eEF1α2, RpL32* using the CFX Manager 3.1 software (Bio-Rad, USA).

## Supplementary Material

Supplementary Figures

Supplementary Table 1
