# Supplementary material for: Evaluation of the geroprotective effects of withaferin A in Drosophila melanogaster
Source: Aging (Albany NY). 2021 Jan 26;13(2):1817–41. doi: 10.18632/aging.202572 (PMC7880378; doi:10.18632/aging.202572)
Supplement: Supplementary Figures [file aging-13-202572-s001.pdf]

SUPPLEMENTARY FIGURES

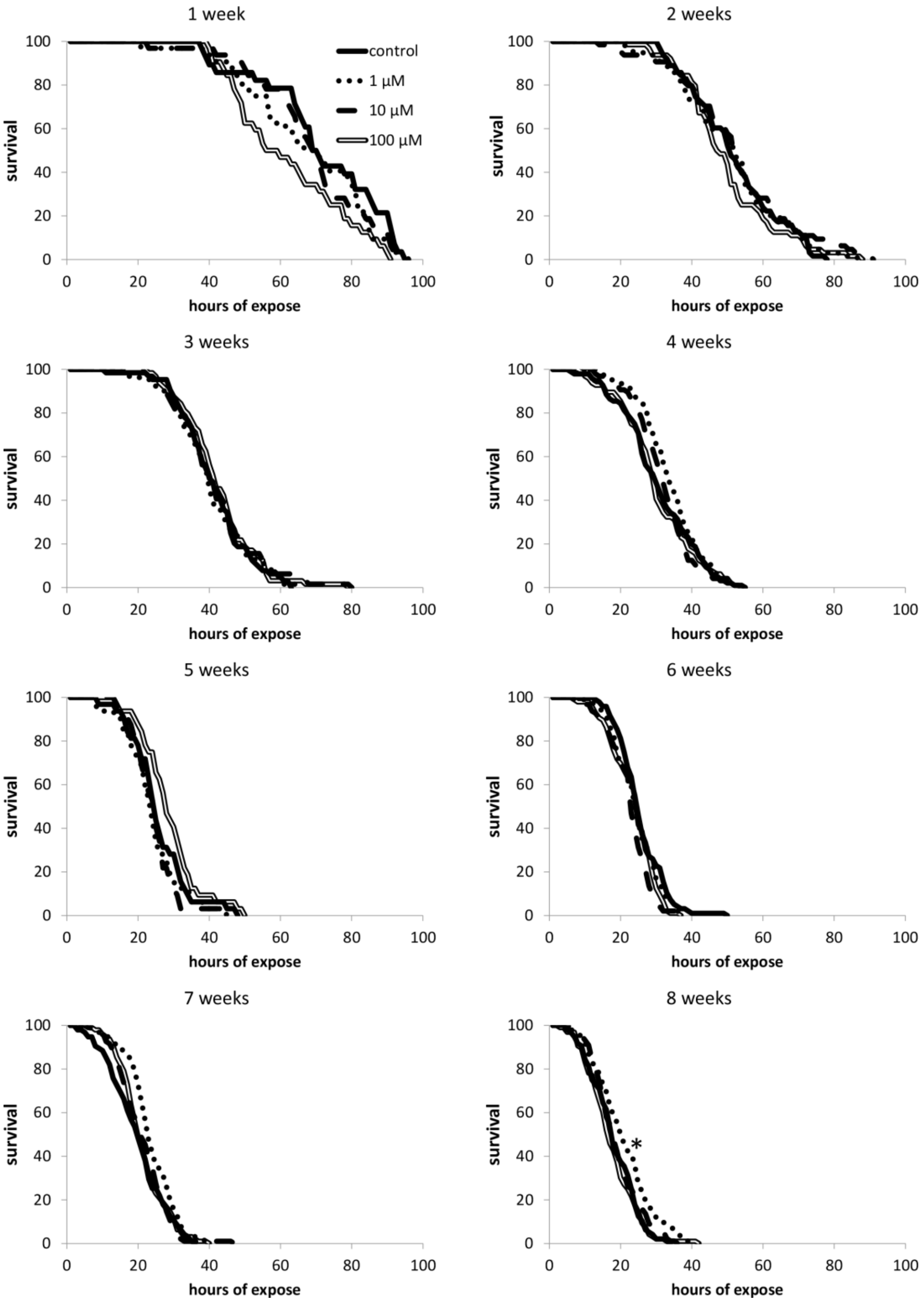

Supplementary Figure 1. The effect of WA treatment on the resistance of *Drosophila melanogaster* to the action of paraquat (oxidative stress) in male at the age of 1 to 8 weeks. Results of three independent repeats are combined. * p <0.01, ** p <0.001, *** p <0.0001.

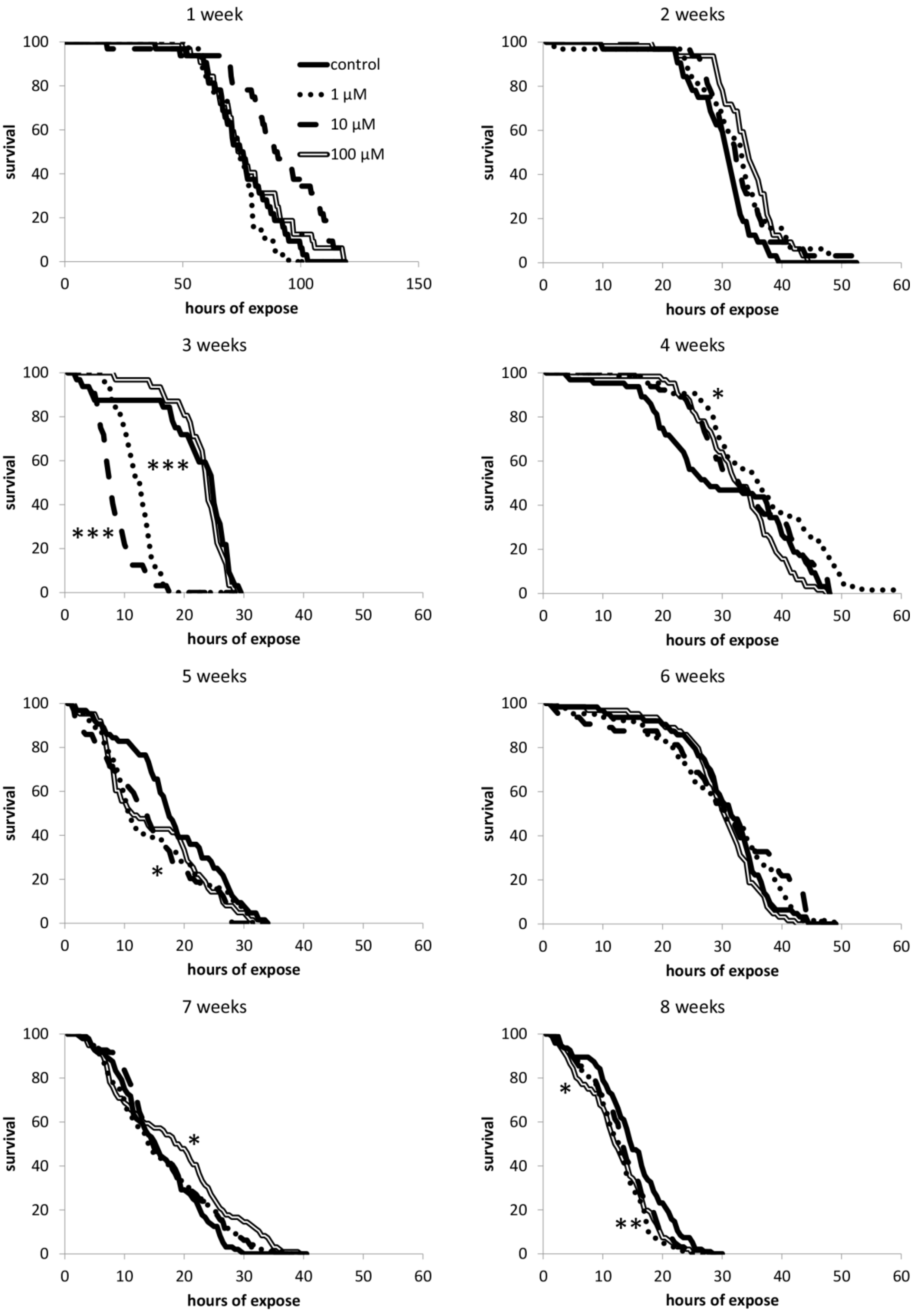

Supplementary Figure 2. The effect of WA treatment on the resistance of *Drosophila melanogaster* to the action of hyperthermia (heat shock) in male at the age of 1 to 8 weeks. Results of three independent repeats are combined. * p <0.01, ** p <0.001, *** p <0.0001.

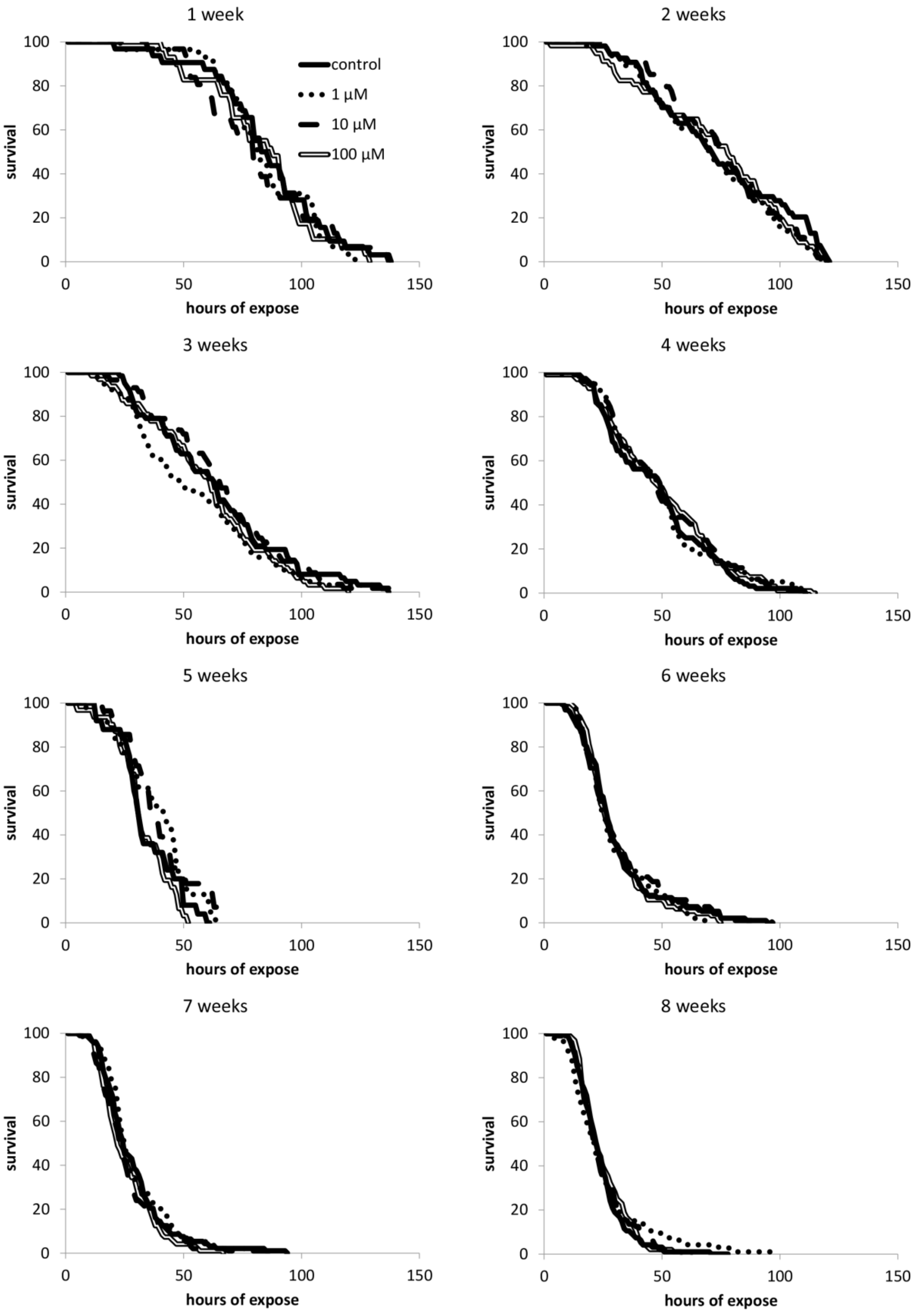

Supplementary Figure 3. The effect of WA treatment on the resistance of *Drosophila melanogaster* to the action of paraquat (oxidative stress) in female at the age of 1 to 8 weeks. Results of three independent repeats are combined. * p <0.01, ** p <0.001, *** p <0.0001.

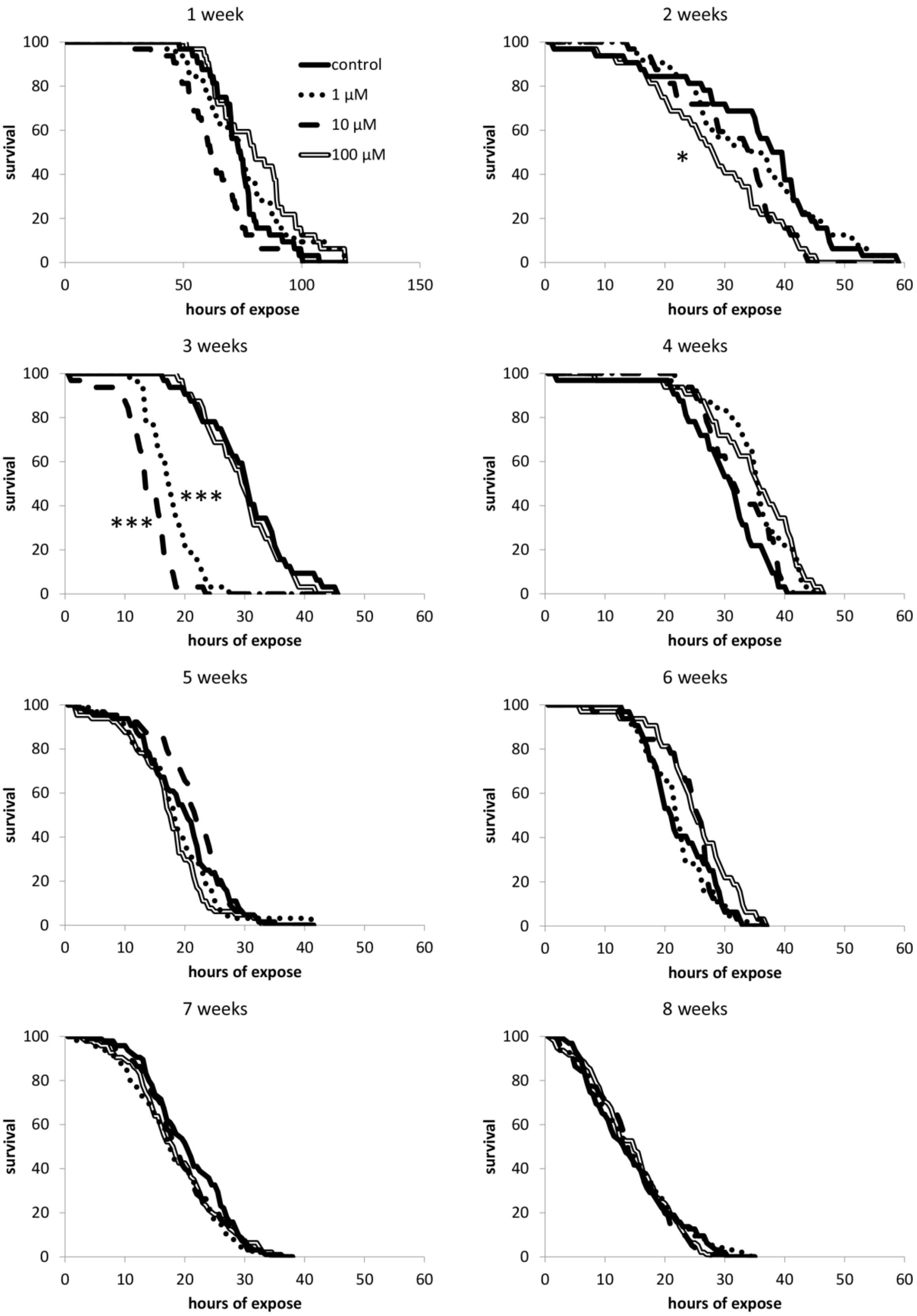

Supplementary Figure 4. The effect of WA treatment on the resistance of *Drosophila melanogaster* to the action of hyperthermia (heat shock) in female at the age of 1 to 8 weeks. Results of three independent repeats are combined. * p <0.01, ** p <0.001, *** p <0.0001.
