# Supplementary material for: Evaluation of the geroprotective effects of withaferin A in Drosophila melanogaster
Source: Aging (Albany NY). 2021 Jan 26;13(2):1817–41. doi: 10.18632/aging.202572 (PMC7880378; doi:10.18632/aging.202572)
Supplement: Supplementary Table 1 [file aging-13-202572-s002.pdf]

SUPPLEMENTARY TABLE

Supplementary Table 1. Role of studied genes in determining the stress response.

Target gene	Characteristics	Effects
Catalase (*Cat*)	Enzymes catalyzes the decomposition of hydrogen peroxide in cells [1].	Is involved in response to photooxidative [2], endoplasmic reticulum [3], oxidative stresses [4] and to hydrogen peroxide stimulus [5]. Overexpression of human catalase, which targets mitochondria, increases life span in *Mus musculus* by about 20% [6].
frataxin (*fh)*	Protein essential for iron-sulfur clusters synthesis, which are necessary for the production of ATP by the respiratory chain, as well as in other biological processes such as steroidogenesis [7–9].	Is involve in response to oxidative stress [7], to hydrogen peroxide stimulus [10], to iron ion homeostasis [9] and to the hypoxia-induced response [11]. Overexpression of frataxin in mitochondria increases the antioxidant capacity, resistance to oxidative stress, and life span in *Drosophila melanogaster* females [12].
Growth arrest and DNA damage-inducible 45 (*Gadd45)*	Participates in the regulation of the cell cycle and oviposition; required to activate MAPKKK, JNK activity [8, 13].	Is involved in response to oxidative, thermal and genotoxic stresses [14, 15]. Overexpression of the *D-Gadd45* gene in the nervous system leads to a significant increase in the life span in *Drosophila melanogaster* [16].
Heat shock protein 68 (*Hsp68*)	Protein necessary for response to temperature or stressing cell stimuli [17].	Is involved to starvation stimulus [18]. Overexpression in somatic cells led to an increase in the average life span of *Drosophila melanogaster* by 20% [18, 19].
Heat shock protein 83 (*Hsp83*)	Protein necessary for response to temperature or stressing cell stimuli [17] orthologous to human HSP90 gene (https://www.ncbi.nlm.nih.gov/gene/38389).	Is involved in response to heat, oxidative stresses and ionizing radiation [20–22], also in regulation of circadian sleep/wake cycle [23].
Ku80 (*Ku80)*	Ku70/Ku80 heterodimer, or Ku, is the central component of the nonhomologous end joining (NHEJ) pathway of double strand break repair [24]. Orthologous to human XRCC5 gene (https://www.ncbi.nlm.nih.gov/gene/7520).	Is involved in gamma radiation and X-ray responses [8, 25]. Deletion of Ku80 leads to signs of premature aging such as osteopenia, atrophic skin, hepatocellular degeneration, and age-related mortality in *Mus musculus* [26]. Also, *Mus musculus* showed a decrease in life span by 40% [27].
Peroxiredoxin V (*PrxV*)	Encodes an atypical member of the thiol-specific peroxidase family, which form intramolecular disulfide bonds during the catalytic cycle [28].	Is involved in response to oxidative stress, cell redox homeostasis and hydrogen peroxide catabolic process [8, 29].Overexpression of PrxV caused an increase in the average and median life span in *Drosophila melanogaster* under normal conditions. Against the dPrxV mutants (- / -) were more susceptible to oxidative stress, had a higher incidence of apoptosis, and a shorter average life span [30].

1. Chelikani P, Fita I, Loewen PC. Diversity of structures and properties among catalases. Cell Mol Life Sci. 2004; 61:192–208. https://doi.org/10.1007/s00018-003-3206-5 PMID:14745498

2. Orlandi VT, Martegani E, Bolognese F. Catalase a is involved in the response to photooxidative stress in pseudomonas aeruginosa. Photodiagnosis Photodyn Ther. 2018; 22:233–40. https://doi.org/10.1016/j.pdpdt.2018.04.016 PMID:29709605

3. Wang L, Zeng X, Ryoo HD, Jasper H. Integration of UPRER and oxidative stress signaling in the control of intestinal stem cell proliferation. PLoS Genet. 2014; 10:e1004568. https://doi.org/10.1371/journal.pgen.1004568 PMID:25166757

4. Ershova OA, Bairova TA, Kolesnikov SI, Kalyuzhnaya OV, Darenskaya MA, Kolesnikova LI. Oxidative stress and catalase gene. Bull Exp Biol Med. 2016; 161:400–03. https://doi.org/10.1007/s10517-016-3424-0 PMID:27496033

5. Sun J, Tower J. FLP recombinase-mediated induction of Cu/Zn-superoxide dismutase transgene expression can extend the life span of adult Drosophila melanogaster flies. Mol Cell Biol. 1999; 19:216–28. https://doi.org/10.1128/mcb.19.1.216 PMID:9858546

6. Schriner SE, Linford NJ, Martin GM, Treuting P, Ogburn CE, Emond M, Coskun PE, Ladiges W, Wolf N, Van Remmen H, Wallace DC, Rabinovitch PS. Extension of murine life span by overexpression of catalase targeted to mitochondria. Science. 2005; 308:1909–11. https://doi.org/10.1126/science.1106653 PMID:15879174

7. Navarro JA, Ohmann E, Sanchez D, Botella JA, Liebisch G, Moltó MD, Ganfornina MD, Schmitz G, Schneuwly S. Altered lipid metabolism in a Drosophila model of Friedreich's ataxia. Hum Mol Genet. 2010; 19:2828–40. https://doi.org/10.1093/hmg/ddq183 PMID:20460268

8. Gaudet P, Livstone MS, Lewis SE, Thomas PD. Phylogenetic-based propagation of functional annotations within the gene ontology consortium. Brief Bioinform. 2011; 12:449–62. https://doi.org/10.1093/bib/bbr042 PMID:21873635

9. Navarro JA, Botella JA, Metzendorf C, Lind MI, Schneuwly S. Mitoferrin modulates iron toxicity in a Drosophila model of Friedreich's ataxia. Free Radic Biol Med. 2015; 85:71–82. https://doi.org/10.1016/j.freeradbiomed.2015.03.014 PMID:25841783

10. Anderson PR, Kirby K, Orr WC, Hilliker AJ, Phillips JP. Hydrogen peroxide scavenging rescues frataxin deficiency in a Drosophila model of Friedreich's ataxia. Proc Natl Acad Sci USA. 2008; 105:611–16. https://doi.org/10.1073/pnas.0709691105 PMID:18184803

11. Guccini I, Serio D, Condò I, Rufini A, Tomassini B, Mangiola A, Maira G, Anile C, Fina D, Pallone F, Mongiardi MP, Levi A, Ventura N, et al. Frataxin participates to the hypoxia-induced response in tumors. Cell Death Dis. 2011; 2:e123. https://doi.org/10.1038/cddis.2011.5 PMID:21368894

12. Runko AP, Griswold AJ, Min KT. Overexpression of frataxin in the mitochondria increases resistance to oxidative stress and extends lifespan in drosophila. FEBS Lett. 2008; 582:715–19. https://doi.org/10.1016/j.febslet.2008.01.046 PMID:18258192

13. Peretz G, Bakhrat A, Abdu U. Expression of the drosophila melanogaster GADD45 homolog (CG11086) affects egg asymmetric development that is mediated by the c-Jun N-terminal kinase pathway. Genetics. 2007; 177:1691–702. https://doi.org/10.1534/genetics.107.079517 PMID:18039880

14. Liebermann DA, Hoffman B. Gadd45 in the response of hematopoietic cells to genotoxic stress. Blood Cells Mol Dis. 2007; 39:329–35. https://doi.org/10.1016/j.bcmd.2007.06.006 PMID:17659913

15. Moskalev A, Plyusnina E, Shaposhnikov M, Shilova L, Kazachenok A, Zhavoronkov A. The role of D-GADD45 in oxidative, thermal and genotoxic stress resistance. Cell Cycle. 2012; 11:4222–41. https://doi.org/10.4161/cc.22545 PMID:23095639

16. Plyusnina EN, Shaposhnikov MV, Moskalev AA. Increase of drosophila melanogaster lifespan due to D-GADD45 overexpression in the nervous system. Biogerontology. 2011; 12:211–26. https://doi.org/10.1007/s10522-010-9311-6 PMID:21153055

17. De Maio A. Heat shock proteins: facts, thoughts, and dreams. Shock. 1999; 11:1–12. https://doi.org/10.1097/00024382-199901000-00001 PMID:9921710

18. Biteau B, Karpac J, Supoyo S, Degennaro M, Lehmann R, Jasper H. Lifespan extension by preserving

proliferative homeostasis in drosophila. PLoS Genet. 2010; 6:e1001159. https://doi.org/10.1371/journal.pgen.1001159 PMID:20976250

19. Wang MC, Bohmann D, Jasper H. JNK signaling confers tolerance to oxidative stress and extends lifespan in drosophila. Dev Cell. 2003; 5:811–16. https://doi.org/10.1016/s1534-5807(03)00323-x PMID:14602080

20. Neal SJ, Karunanithi S, Best A, So AK, Tanguay RM, Atwood HL, Westwood JT. Thermoprotection of synaptic transmission in a drosophila heat shock factor mutant is accompanied by increased expression of Hsp83 and DnaJ-1. Physiol Genomics. 2006; 25:493–501. https://doi.org/10.1152/physiolgenomics.00195.2005 PMID:16595740

21. Landis G, Shen J, Tower J. Gene expression changes in response to aging compared to heat stress, oxidative stress and ionizing radiation in drosophila melanogaster. Aging (Albany NY). 2012; 4:768–89. https://doi.org/10.18632/aging.100499 PMID:23211361

22. Singh MP, Reddy MM, Mathur N, Saxena DK, Chowdhuri DK. Induction of hsp70, hsp60, hsp83 and hsp26 and oxidative stress markers in benzene, toluene and xylene exposed Drosophila melanogaster: role of ROS generation. Toxicol Appl Pharmacol. 2009; 235:226–43. https://doi.org/10.1016/j.taap.2008.12.002 PMID:19118569

23. Shaw PJ, Franken P. Perchance to dream: solving the mystery of sleep through genetic analysis. J Neurobiol. 2003; 54:179–202. https://doi.org/10.1002/neu.10167 PMID:12486704

24. Postow L, Ghenoiu C, Woo EM, Krutchinsky AN, Chait BT, Funabiki H. Ku80 removal from DNA through double strand break-induced ubiquitylation. J Cell Biol. 2008; 182:467–79. https://doi.org/10.1083/jcb.200802146 PMID:18678709

25. Zhang C, Hong Z, Ma W, Ma D, Qian Y, Xie W, Tie F, Fang M. Drosophila UTX coordinates with p53 to regulate ku80 expression in response to DNA damage. PLoS One. 2013; 8:e78652. https://doi.org/10.1371/journal.pone.0078652 PMID:24265704

26. Vogel H, Lim DS, Karsenty G, Finegold M, Hasty P. Deletion of Ku86 causes early onset of senescence in mice. Proc Natl Acad Sci USA. 1999; 96:10770–75. https://doi.org/10.1073/pnas.96.19.10770 PMID:10485901

27. Holcomb VB, Rodier F, Choi Y, Busuttil RA, Vogel H, Vijg J, Campisi J, Hasty P. Ku80 deletion suppresses spontaneous tumors and induces a p53-mediated DNA damage response. Cancer Res. 2008; 68:9497–502. https://doi.org/10.1158/0008-5472.CAN-08-2085 PMID:19010925

28. Knoops B, Goemaere J, Van der Eecken V, Declercq JP. Peroxiredoxin 5: structure, mechanism, and function of the mammalian atypical 2-Cys peroxiredoxin. Antioxid Redox Signal. 2011; 15:817–29. https://doi.org/10.1089/ars.2010.3584 PMID:20977338

29. Poole LB, Hall A, Nelson KJ. Overview of peroxiredoxins in oxidant defense and redox regulation. Curr Protoc Toxicol. 2011; Unit7.9. https://doi.org/10.1002/0471140856.tx0709s49 PMID:21818754

30. Radyuk SN, Michalak K, Klichko VI, Benes J, Rebrin I, Sohal RS, Orr WC. Peroxiredoxin 5 confers protection against oxidative stress and apoptosis and also promotes longevity in drosophila. Biochem J. 2009; 419:437–45. https://doi.org/10.1042/BJ20082003 PMID:19128239
